# “You're Really Gonna Kick Us All Out?” Sustaining Safe Spaces for Community-Based HIV Prevention and Control among Black Men Who Have Sex with Men

**DOI:** 10.1371/journal.pone.0141326

**Published:** 2015-10-22

**Authors:** Jonathan Garcia, Caroline Parker, Richard G. Parker, Patrick A. Wilson, Morgan M. Philbin, Jennifer S. Hirsch

**Affiliations:** 1 School of Biological and Population Health Sciences, College of Public Health and Human Sciences, Oregon State University, Corvallis, Oregon, United States; 2 Department of Sociomedical Sciences, Mailman School of Public Health, Columbia University, New York, New York, United States; 3 HIV Center for Clinical and Behavioral Studies, New York State Psychiatric Institute, New York, New York, United States; China Medical University, CHINA

## Abstract

Black men who have sex with men (BMSM) experience among the highest rates of HIV infection in the United States. We conducted a community-based ethnography in New York City to identify the structural and environmental factors that influence BMSMs vulnerability to HIV and their engagement with HIV prevention services. Methods included participant observation at community-based organizations (CBOs) in New York City, in-depth interviews with 31 BMSM, and 17 key informant interviews. Our conceptual framework shows how creating and sustaining safe spaces could be a critical environmental approach to reduce vulnerability to HIV among BMSM. Participant observation, in-depth and key informant interviews revealed that fear and mistrust characterized men’s relation to social and public institutions, such as churches, schools, and the police. This fear and mistrust created HIV vulnerability among the BMSM in our sample by challenging engagement with services. Our findings suggest that to be successful, HIV prevention efforts must address these structural and environmental vulnerabilities. Among the CBOs that we studied, “safe spaces” emerged as an important tool for addressing these environmental vulnerabilities. CBOs used safe spaces to provide social support, to address stigma, to prepare men for the workforce, and to foster a sense of community among BMSM. In addition, safe spaces were used for HIV and STI testing and treatment campaigns. Our ethnographic findings suggest that safe spaces represent a promising but so far under-utilized part of HIV prevention infrastructure. Safe spaces seem integral to high impact comprehensive HIV prevention efforts, and may be considered more appropriately as part of HIV capacity-building rather than being nested within program-specific funding structures.

## Introduction

From 2008–2010, black men who have sex with men (BMSM) represented only 2% of the US population but approximately 75% of new HIV infections [1]. In 2011, 66.1% of all new HIV diagnoses in New York City were among black men, of which 56.2% were MSM [2]. Epidemiological and social research has identified structural and environment factors that deter BMSM from engaging with health services, testing for HIV, and that challenge adherence to treatment and care [3,4]. Principal environmental determinants of health disparities among BMSM include neighborhood violence [5,6], discriminatory policing practices (which drive social stigma and exacerbate disenfranchisement) [7–9], hate crime against LGBT persons [10,11], and being thrown out of their homes when they disclose their sexuality or have feminine gender performance [12–14]. Thus, an environmental approach to HIV prevention is particularly important for BMSM, among whom HIV remains a public health emergency.

In the context of recent advances in biomedical HIV prevention strategies, community-based engagement continues to be crucial for addressing environmental vulnerabilities in rolling out emerging prevention technology [15]. However, there is little research that directly investigates organizational capacity to generate community-ownership and sustain mobilization and advocacy for access to emerging biomedical prevention strategies [15]. Although recent research has proposed combining structural, environmental, behavioral, and biomedical prevention as part of comprehensive prevention for the most at-risk groups (i.e., high-impact prevention) [16–19], we could not identify any studies that directly discuss how community-based engagement can be used to advance high-impact prevention for BMSM.

**Fig 1:**
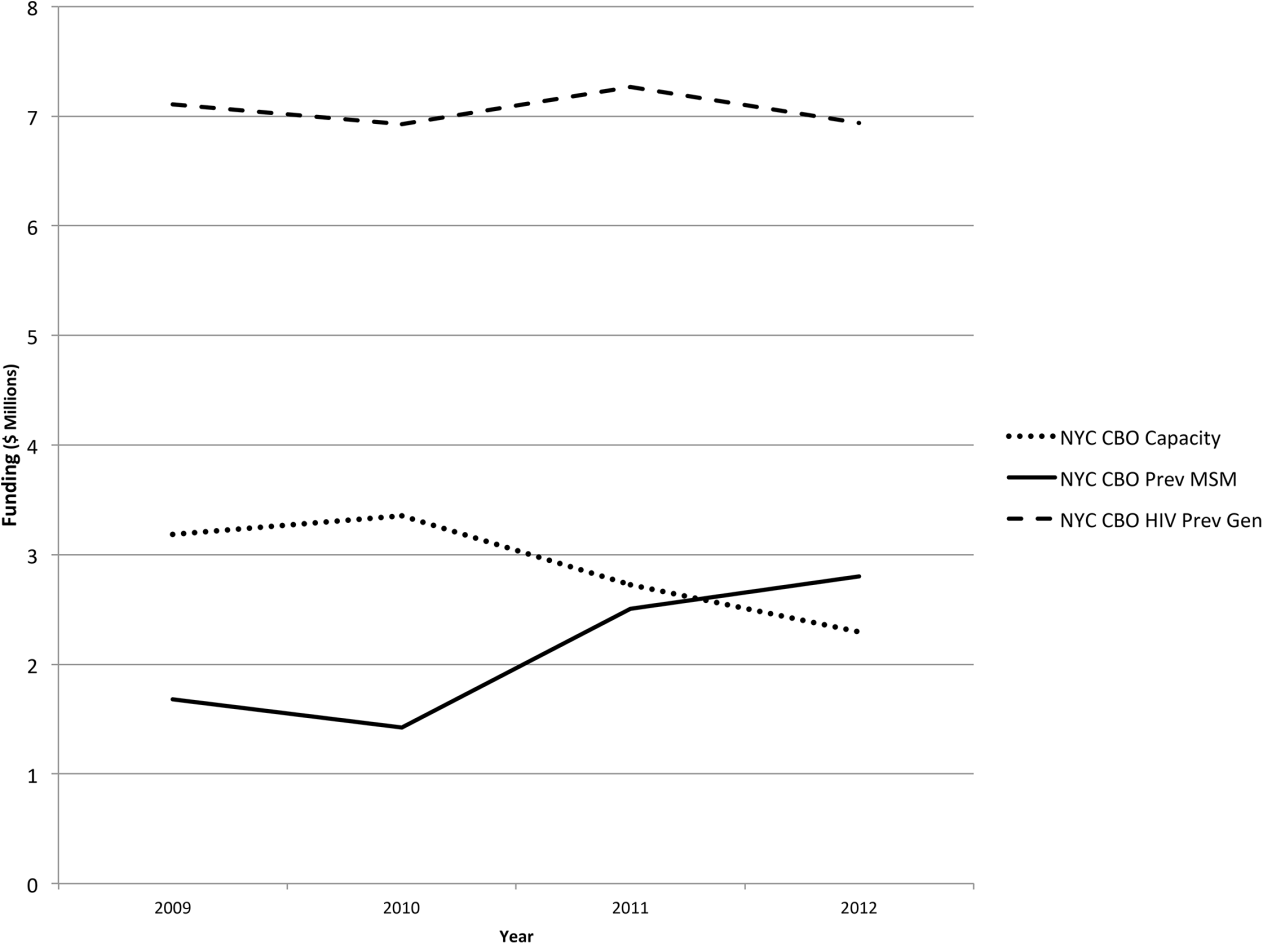
CDC Funding for HIV Prevention in CBOs, New York City Metropolitan Area, 2009–2012*. * Source: Division of HIV/AIDS Prevention (DHAP) HIV Funding Awards by State and Dependent Area, Funding 2009–2012, available at http://www.cdc.gov/hiv/policies/funding/index.html.

Community-based approaches to HIV prevention are an essential part of the National HIV/AIDS Strategy [20]. However, interventions and research agendas currently emphasize individual-level biomedical prevention approaches without sufficient consideration for community-led responses [15,21–24]. Of the total federal budget for HIV and AIDS for 2015, domestic HIV prevention constituted 3% of the overall budget [25]. Within this, HIV prevention funding is geared towards individual-level biomedical prevention (testing, condom distribution, surveillance), and from 2010–2012, community-based organizations in New York City experienced funding cuts of 32% for capacity-building projects and training (see Fig 1).

In 2014 the Centers for Disease Control and Prevention (CDC) awarded $115 million over 5 years to 21 organizations, as part of its Capacity Building Assistance (CBA) program [26,27]. Explicit emphasis was placed on behavioral interventions and proven cost-saving measures [26], a negative consequence of which was that no Black civil society organizations were granted funding [27,28]. According to the Black AIDS Institute, these criteria for allocating funding have effectively “locked out” Black civil society organizations for the next five years [27]. The lack of funding mechanisms that provide funding specifically for infrastructure at these organizations may present problems for implementing culturally appropriate projects for BMSM. For instance, researchers with the Translating Research into Practice (TRIP) Project recently conducted a national evaluation of MPowerment (a CDC evidence-based intervention to prevent HIV), finding that having a safe space was an integral facilitator of programmatic fidelity [29]. However, they also noted that these spaces are not explicitly funded by any mechanism when the CBO sought to implement MPowerment. In this article, we call for heightening efforts to sustain CBOs and in particular to sustain safe spaces, as an essential form of HIV prevention capacity building that is vital for developing culturally appropriate, community-led approaches.

To explore the potential of community-based engagement for BMSM for HIV prevention, in this article we present the findings from a community-based ethnographic study that worked with BMSM and HIV service providers in New York City. This research sought to identify the structural and environmental factors that influence BMSMs engagement with HIV prevention services, and in particular BMSMs engagement with HIV prevention. “Safe spaces” emerged as central to the work of some community-based organizations (CBOs) that we worked with, and our ethnographic findings suggest that the creation and community monitoring of safe spaces should be integral to comprehensive HIV prevention efforts. First, we describe the structural and environmental factors that generate HIV vulnerability among BMSM. Then, we describe the centrality of safe spaces for addressing key aspects of HIV vulnerability, drawing upon participant observation at CBOs as well as interviews with service providers. Finally, we examine the facilitators and barriers that clients and service providers face in their efforts to maintain safe spaces, and we provide recommendations regarding the role of safe spaces for community-led HIV prevention.

## Methods

Data collection took place between June 2013 and May 2014 and consisted of: 11 months of participant observation, in-depth interviews with 31 BMSM (3, 90-minute interviews per individual), and 17 60-minute interviews with community stakeholders. Participant observation took place twice per week over the course of 11 months in spaces recommended by our Community Advisory Board or that emerged in interviews. Participant observation consisted of “community observation” and “short term observation” with in-depth interviewees. In community observation, we participated in weekly community events and public forums (e.g., weekly happy hour with black LGBT groups, mobilization discussions at community-based organizations). In “short term observation,” the lead ethnographer (first author) accompanied 5 in-depth interviewees to places where the in-depth interviewees agreed to invite the ethnographer (e.g., private house parties, gay family dinners, police precincts, public cruising in parks among other situations). Short-term observation was particularly useful for documenting the private domain, as well as triangulating interview data from in-depth interviewees that also invited the lead ethnographer to hang out. Participant observation was recording in jottings, fieldnotes, maps, and analytic memos. The lead ethnographer’s own subjectivities and sensibilities as a gay Latino man may have facilitated ‘hanging out’ with other men who were also racial-ethnic and sexual minorities. The first author conducted face-to-face interviews with BMSM and community stakeholders. In-depth interviews were used to collect narrative in the domains described in Table 1. Men’s past and current life experiences, stories, and particular examples were elicited to describe how social and economic situations contextualize risk practices and health promotion opportunities. All in-depth interviewees were aged 15 and older, born as and identified as male, and reported having had anal or oral sex with a man within the past year. Recruitment consisted of outreach and advertising in bars, clubs, community health centers, community-based organizations, and the Internet. Key informant interviews with community stakeholders explored sources of contextual vulnerability, organizational facilitators and barriers to implementing HIV prevention, institutional knowledge, and work specifically focused on BMSM and PrEP. We worked with two Community Advisory Boards (a client CAB and a provider CAB) to identify community stakeholders (key informants) that have been active in addressing HIV risk among BMSM in New York City. All participants were read a verbal informed consent form. Because participants were being asked sensitive questions that could potentially reveal information about sex work and drug use, verbal consent provided a higher level of confidentiality than written consent. Participants were made aware that a Certificate of Confidentiality granted by the National Institute of Mental Health protected data collected. Their understanding was assessed with a series of follow-up questions about the content of the consent form. This assessment of understanding was audio-recorded and data collection began following participants’ recorded declaration of verbal consent. All procedures described here were approved by the Columbia University Medical Center Institutional Review Board.

**Table 1:** Methods.

Data collection method	Specific data elicited	Sample description
**Participant**	Organizational behavior, group composition,	11 months of observation
**Observation**	ways people discuss sexuality, race, gender, age, class; /interaction, spoken rules of conduct and implicit cultural norms expressed, enforced, followed and navigated.	in: private spaces (homes, parties); public spaces (parks, streets, events); virtual spaces (chat rooms, blogs, social media); and institutions (community organizations, health centers, religious institutions).
**Key Informant Interviews**	Organizational mission; role in organization/group/community; knowledge and attitudes about black MSM, HIV vulnerability, institutions and networks available to Black MSM; views on PrEP and other for HIV/STI prevention	17 informants, including 2 physicians; 3 mental health providers; 4 community organization program administrators; 5 outreach workers; 3 community mobilizers
**In-depth Interviews**	Session 1: History of family relations, coming of age, education, housing, making money, friends; community, recreation	31 participants
	Session 2: Sexual history, including desire, casual and steady relations, sexual identity and racial identity	
	Session 3: Perceptions of health and risk; practices and attitudes about medications and seeking health services; knowledge and attitudes about HIV prevention and PrEP	

Interviews were digitally-recorded and transcribed verbatim. Interview data and fieldnotes were analyzed and triangulated between and across cases using Atlas.ti 7.0 qualitative software [30]. The social ecological framework was used to analyze multiple levels of vulnerability that BMSM encounter, on the individual, interpersonal, institutional, and structural levels [31,32]. In the first step of data analysis we constructed a codebook from data groups based on domains derived from interview guides. Through iterative open coding and research team discussions we added new categories of data to this codebook. The first and second authors double-coded 20% of all data and met weekly to reconcile discrepancies that appeared through open coding. The coders arrived at an inter-coder agreement greater than 80%, and used the resulting codebook to complete coding data. Subsequently, in a second cycle of data organization, conceptual patterns and theoretical themes emerged from the data were also coded. Coding interviews and fieldnotes using the same codes facilitated data triangulation. Here we draw on several code families, including “community infrastructure,” “community engagement,” “community identity,” “violence,” “stigma/discrimination,” “gender performance,” “sexual identity,” “community-based organizations,” “engagement in health services,” as well as from open codes such as “safe spaces” that emerged organically from the data. Community Advisory Boards and a local AIDS Service Organization provided feedback in several instances throughout the data analysis process by reading data analysis reports and reflecting on the validity of our findings in their communities or service organizations.

## Results

**Fig 2:**
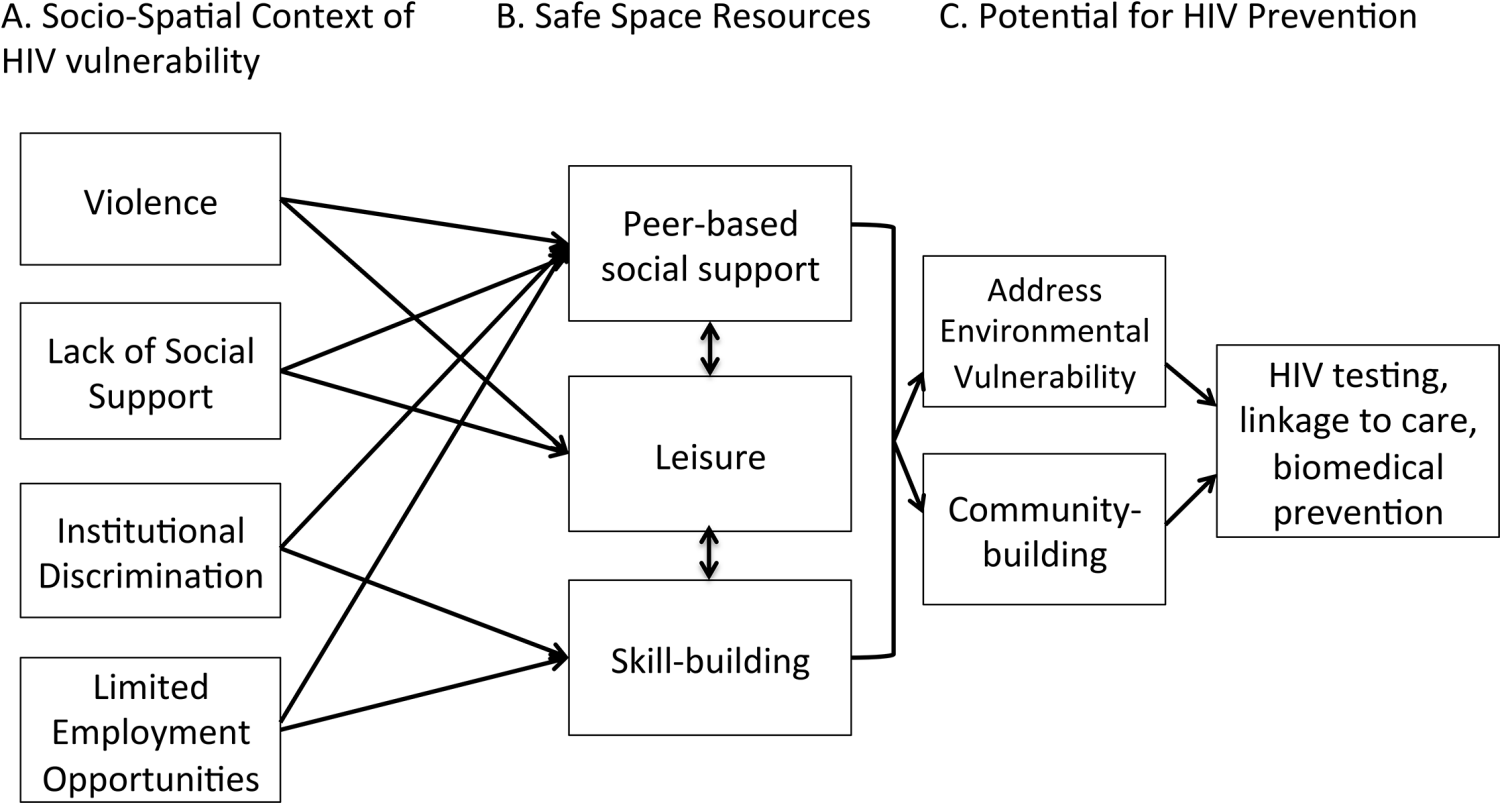
Safe Spaces to address socio-spatial HIV vulnerabilities and promote prevention.

Of the 31 BMSM in our sample, the mean age was 29, with 17 men 15–24 of age and 14 men 25 and older. More than half of the men identified as gay or same-gender loving (N = 18). Other men identified as straight, discreet, bisexual, or preferred no sexual identity (N = 13). Just over half of the men in our sample were either homeless (N = 5) or had unstable housing (N = 11). Nearly half of them were unemployed (N = 15) and 8 were part-time/temporary. Seventeen men were on Medicaid, 9 were uninsured, and 5 had private insurance (S1 File). Approximately three-quarters of the sample accessed health services at community-based clinics, primarily for HIV testing and not for routine check-ups. Many men experienced racial profiling by law enforcement (N = 18) and reported a mistrust of medical institutions or physicians (N = 17). The following sections outline the conceptual framework that emerged from our findings (see Fig 2). This figure illustrates the structure of our findings.

### A. Socio-spatial context of HIV vulnerability

#### Violent Spaces

In interviews, men were asked about their feelings of safety and danger in their daily lives. Feeling unsafe in certain public spaces was a common theme among the men in our sample, and men frequently referred to instances of violence in public spaces. Some men had experienced violence first-hand, while others referred to friends’ experiences. Nearly all men made reference to incidents of LGBT hate crime reported in national and local media. Men described the fear of being attacked as linked to the ethnic composition of the neighborhood. Several commented that neighborhoods with a higher concentration of immigrants from the Caribbean were more dangerous for non-heterosexuals. These men noted that they felt Brooklyn was the most dangerous because of violent homophobia they witnessed. Many mentioned the need for more community-engagement to address violence against LGBT persons, and that a lower concentration of LGBT CBOs in Brooklyn and the Bronx perpetuated perceived danger. As a 22-year-old man explained, “We need more LGBT groups working in Brooklyn because more black gay men moving there.” Perceiving hate crime to be a major problem, he further recounted:

Oh the Bronx is dangerous, Brooklyn is dangerous, oh, you know, gay bashing here and there all over the place, you know… Yeah, like I’ve heard more slurs; gay friends or associates of mine getting jumped or getting into fights, just all out brawls in Brooklyn.

An outreach worker at a community-clinic serving mostly young MSM of color claimed that the surrounding neighborhood created hostility for “feminine” clients: “transwomen and feminine men were harassed by neighborhood men who call them faggots… Sometimes if they’re feminine, men will make cat calls late at night to harass them or because they want sex.” A 27-year-old same-gender loving man explained a conversation he had with his mother when he disclosed his sexuality in which she was primarily concerned about femininity as “something extra that puts me in danger for no reason.” He continued:

But I told her, I was like, "You know, I act pretty normal." I don't really call it normal, but that's the best word to use for it, like I'm not flamboyant, I'm not out in the open, I don't do anything to advertently put myself into danger because of my lifestyle, and stuff like that. I try to refrain from individuals that will put me into that arena….people who are just too loud…I have no problem with you expressing yourself and stuff like that, but I believe that there's always a time and a place for everything, and some people don't understand that.

His narrative highlights how gender conformity is socially policed, as well as how femininity (e.g., being “flamboyant” or “too loud”) is read by others as a sign of homosexuality. Feminine gestures, feminine clothing and having feminine friends, were seen as signs of homosexuality that many of the men in our sample tried to avoid, reflecting a self-consciousness about showing their sexuality in an environment of hostility towards homosexuality and femininity. Most BMSM and key informants expressed that public and commercial spaces were more dangerous for feminine men than for masculine men who could “pass” (as straight).

All the violent stuff that's going on nowadays is more stressful to me, like people crossing the street and beating up people for no reason because they gay or look gay. And that's been on the news quite often. I even told my boyfriend, because I know how feminine he walks and carry himself, "Be careful out there." And when I'm walking with him, I try to be careful too. I wouldn’t walk with him if he was in an area where it’s dangerous–called the projects. (32, no sexual identity)

In in-depth interviews, men emphasized that femininity, or feminine gender performance, made men more likely to be targeted by more masculine MSM.

It’s threatening to be with a feminine man… because you don't want people looking at you in a certain way. Stereotypes are for feminine men. They tend to be loud, obnoxious, ghetto, flamboyant, and just messy. (18, gay)

Several men described witnessing violence against men who were “flamboyant” perpetrated by other MSM, who “use that self-hatred and violence on other [MSM] brothers to protect their image.” In addition, the narratives of several BMSM and evening observations in public parks provided examples of instances in which men expose themselves to violence in public sex settings in parks after they have casual sex. A 31-year-old man, who accompanied the lead ethnographer in participant observation, explained the danger of cruising in the park because some sexually discreet men react violently after sexual encounters.

In some parks, you have to time yourself to a certain degree where you wanna get out at a certain time because cops can't see you … And [men] take the risk of possibly being caught because they feel like they need to do whatever before they go wherever they travel, whether it’s home or to the club or from the club…. And sometimes that’s why it’s very important to kind of being able to assess the situation because… they will hurt you or attack you after. (31, gay)

The risk of violence resulting from “internalized homophobia” in discreet spaces such as parks contributed to context that facilitated “rushed sex” and drug use. In these spaces, men seemed to place higher priority on avoiding arrests by the police than they did on avoiding the other dangers of those spaces (namely, physical violence, sexually transmitted infections from unprotected sex).

Men also described dangers in gay clubs where the clientele was predominantly Black. Although these places were supposed to be safe for gay men, a 26-year-old man told the story of being attacked by older, more masculine gay men in a gay club:

We all fight about not having equal rights because straight people are out here jumping and hate-criming us, but yet we’re in the clubs doing the same thing over petty stuff… And [the attacker was] masculine, you would never be able to tell he’s gay even though he has a boyfriend. So it was a bunch of grown gay men just attacking me. (26, gay)

Several men avoided bars because, as a 22-year-old gay man explained, the night ends in fights with men “throwing bottles” at each other. Thus, men in our sample reported feeling unsafe in a variety of public spaces (e.g., street and parks) and commercial spaces (e.g., bars and clubs). This was related to first-hand experiences of violence as well as perceptions of the threat of violence. Thus, in commercial spaces, such as bars and clubs, as well as in public cruising areas, men felt unsafe because the perpetrators of violent acts were other MSM. Violence was related not only to sexuality but also to gender performance.

#### Discriminatory Institutions

Men reported experiencing institutional stigma and discrimination because of their race, sexuality, socioeconomic class, and gender performance. Participant observation, in-depth and key informant interviews revealed that fear and mistrust characterized men’s relation to social and public institutions, such as law enforcement, churches, schools, and the family. Most straight, discreet, and bisexual men kept their sexuality a secret at churches because of the fear of being rejected and shamed. Overall, the environment of hostility and danger that BMSM faced created mistrust, and social isolation. Negative experiences in schools undermined educational success and limited employment opportunities.

Many men in our sample perceived to have experienced racial discrimination from law enforcement, leading them to feel “persecuted” instead of protected by the police.

“So the police came up and they started just questioning what we were doing, why we were there. And of the people who were there, I felt singled out because I was visibly a person of color and the majority of them were not.” (22, gay)

“The cops stopped me and told me that I resemble a homicide suspect. And they searched me and then they asked me why I had so many singles in my pocket. And I told them that I was a waiter because I make tips. And he asked me what was I doing in this area and this and that. And I don't know. So that was another experience that I had… So my mother told me, ‘You should have asked them what was the homicide suspect? Was he black? So because you’re black you look like him?’” (21, bisexual)

Through participant observation, it became clear that there was a “culture of mistrust” in communities, such as Harlem, where some participants expressed feeling that a police presence was there to “protect the White people gentrifying the hood.” In addition, several in-depth interview participants reflected public dialogue we observed about the use of “condoms as evidence” of sex work.

“Cops believe if you have condoms on you, you’re a sex worker. And we believe mostly in this community if you have condoms on you a lot, if you have condoms all over your house, that you’re a ho and that’s crazy we believe that in this community.” (29, gay)

“I feel that why would you lock someone up for that unless you caught them doing sex work? You know what I mean? Yeah, but they're not, so you just catch them with the condom. That's just really stupid. People need condoms.” (21, gay)

The general mistrust of law enforcement and knowledge that carrying condoms could be used against them in court effectively deterred several participants from carrying condoms.

Some men reported that they no longer attended church because of outspoken preaching against homosexuality, feeling they were not “part of” the community because they were treated as sinners. Many men who continued to attend church struggled to rectify their homosexuality with their religious beliefs, and felt that homosexuality was at odds with their religion. One 20-year-old discreet man who internalized the identity of “the sinner,” explained:

They can put a smile on their face and say, “Hate the sin but love the sinner,” but you really don’t know what they’re thinking…I mean it IS stated in the Bible that God doesn’t agree with homosexuality. The Sodom and Gomorrah reference is used a lot. It’s preached against. (20, discreet)

A psychologist explained that some of his clients continued to go to church to be “flogged” by discriminatory messages resulting in “theological trauma”, and related that some of his clients actively denied their homosexuality at church to “feel like they belong” and thus to maintain social ties with the church. Continuing to attend church services in light of these overwhelmingly negative experiences points to the importance of the church as a cultural institution in many black communities and its emotional impact on BMSM seen as psychological violence.

In addition, several young men reported being bullied at school because of their femininity or because they disclosed their homosexuality, which led to fights, public shaming, and sometimes dropping out. A 17-year-old gay man explained that dropped out in the eighth grade because people made fun of his femininity. He recounted being mocked because of his hand gestures. [people would say,] “look he’s twitching.” Another 17-year-old, gay man recounted that he “got pulled out of school”. He explained that during a period in which he was intensively bullied about his sexuality, he had performed badly in his end of year exams, which culminated in him dropping out in the 11th grade. A 21-year-old bisexual man recounted dropping out because of “difficulties coming out to people” at the same time that he had “difficulties with family members about [his] sexuality.” For many men in our sample, and not just those that dropped out, dealing with bullying, hostile school environments, and with adolescent pressures relating to their sexuality and sexual identity contributed to poor educational outcomes, which had enduring consequences for their later employment opportunities.

Persecution at school was echoed, and for some men exacerbated by, homophobia at home. Men who dropped out of school because of bullying often had unsupportive parents who did not accept their homosexuality, femininity, or their gay friends. Most men reported exposing themselves to danger by running away and staying out all night with other older men to seek comfort because of the lack of social support from their families, especially when they were rejected because of their sexuality. In some instances, this resulted in homelessness. Men who reported being rejected by their families often expressed having problems with self-worth, and a few linked their lack of support at home with “reckless sexual behavior.” A 24-year-old gay man who had a difficult relationship with his mother because of his sexuality rationalized:

I think if my father would have been around, maybe my life would have changed. Maybe I wouldn’t be having sex with men. Maybe that wouldn’t have happened…from drug dealing to sex work and everything. (24, gay)

Many men described how the lack of support from key social institutions, such as churches and their families, reduced their feelings of self-worth and rendered them more vulnerable to HIV, drug use, and limited educational and employment opportunities. A 46-year-old discreet man sums up the kinds of social strains men experienced from homophobic institutions.

Negativity [that] comes with being homosexual from heterosexuals, from religion, from your grandma, from your aunt, from your uncle’s buddy, from your uncle–they could say everything about faggots and lesbians… It’s not supported in the black community; it’s definitely not supported through church.

He went through a period in his youth when he was homeless because he wanted to experiment with homosexuality. During this period, he became isolated from his former peer group, and recounted losing several of his closest friends and becoming isolated. These issues of social isolation, lack of family support, and dropping out of school and running away were a recurrent theme, both among the younger men, many of whom were currently experiencing these social processes of exclusion, as well as among the older men, whose narratives about their difficulties during adolescence echoed those of the younger men. Their shared experience strongly indicates that a lack of social support across the multiple institutions of law enforcement, school, family and church exposed men to a range of negative experiences (e.g., housing insecurity, educational failure, social isolation).

### B. Safe spaces as resources for community engagement and institutional capacity

Across the 17 community stakeholders that we interviewed and the eight organizations at which we conducted participant observation, and in conversation with our provider and client community advisory boards, safe spaces emerged as an important dimension of their HIV prevention work. In the NGOs/CBOs visited, safe spaces were a central element of men’s support groups. Within safe spaces, activities and services ranged from group discussions about social justice and issues around sexuality and sexual health, to resume-writing and tutoring services and leisurely activities. Drawing on content analysis of interviews and field notes, a working definition of “safe space” emerged as outlined below.

1. Safe spaces promote empowerment and community mobilization against stigma, discrimination and violence.
2. Safe spaces enable human development by providing skill-building opportunities to those who experience marginalization from educational and work environments.
3. Safe spaces promote supportive social norms and peer networks through a range of leisurely activities that are culturally relevant.

Community stakeholders felt that these safe spaces were essential, not simply as physical spaces with rules that offered an environment free of the stigma and discrimination and as alternative “hang-outs” to the dangerous contexts and situations described above, but also in their role in facilitating social support, leisure-time and recreational activities. Home cooked meals that staff brought to potluck dinners, tutoring sessions, video game nights (“Gay-mers Night”), and dance sessions were some of the forms of leisure we observed at CBOs. Safe spaces created a community of people who could provide mutual social support and organize activities—which several CBO coordinators found essential to recruiting and retaining clients for services and continuous engagement with healthcare.

Interviews revealed that support groups were structured as surrogate homes or families for men to gain social support that they otherwise lacked. HIV CBO administrators and outreach workers drew on ideas of the “home,” the “living room” and the family to describe how clients were supported in these spaces, and said that one of the reasons their clients are drawn into these spaces was because they allowed “*people freedom to be themselves and to really feel like a community*, *to feel like a family”*. One CBO administrator described the safe space that his organization provided thus:

A safe space is where you come. You're not judged. It’s free. No one’s going to hurt you. We have food, clothing, shower, anything. Like it’s a safe haven. Like if you don't have a home, you have a safe space to come. (Outreach worker/HIV tester)

Men’s support groups were often called “brotherhoods” because men could “support each other as males” to “fill a void,” usually because they were not accepted as homosexual by their biological families. Through attendance and participation in these support groups, men formed close social ties, which came to be understood as alternative families, as one man explained.

I guess from your family standpoint, you have a brother and they're not gonna be perfect, but they're gonna always be around and meet you half the time. Well, you're gonna lift them up and they're gonna lift you up. You know, you're gonna look out for each other. (17, gay)

By providing a space for leisure, safe spaces enabled discussions about public health messages related to black male sexuality, perceived HIV risk, and perceptions about being socially targeted as dangerous because of their race, gender and socioeconomic status. Together in these spaces, men reflected on their social standing and how their self-perception affected their negative outlook about acquiring HIV. Through participant observation in these brotherhoods, we witnessed the intergenerational exchange of knowledge where men expressed “not feeling boxed up into categories as black gay men, being able to reject rather than accept multiple stereotypes.” Not being “boxed in” also meant collectively developing and articulating a critique of public health discourse that “equates being a black gay man with being at-risk or promiscuous”, which men discussed as stigmatizing. The safe space provided protection from discrimination and an environment that was mutually supportive for confronting stigma related to sexuality, race, and HIV. Many men agreed that these were spaces where they could offer each other “mutual support to confront common problems.” As a 31-year-old, gay man explained, these problems included HIV, but also:

It was a space where black men would meet and socialize with one another. We’d have these conversations, anything from politics or what’s happening specifically in the black gay or black homosexual community and just about being a bunch of brothers. And it age-varied. I think the oldest may have been 70, and the youngest may have been 18 or 19.

Thus, both BMSM and key informants referred to the “safe space” as a kind of surrogate family, which provided the infrastructure for emotional support to undercut experiences with danger and homophobia in men’s neighborhoods, families, churches, schools, among other spaces previously described.

### C. Potential for HIV Prevention and Health Promotion

Based on these findings, Fig 2 reflects our understanding of how participating in peer-led groups (e.g., brotherhoods), leisure-time activity, and skill-building exercises in dedicated safe spaces can affect men’s self-worth and their sense of belonging to (“being a part of”) a community. A 17-year-old bisexual man described a theatre program based at an HIV CBO as his “peaceful place;” that was where he built his “self-confidence.” He explains that “all the stories in the plays and the scenes are based off us.” Belonging to this group helped him understand and “rewrite” his battle with being bullied in school due to his sexuality. In addition, he claimed:

We do training, as in we do exercises, team building, skill building. We learn how to work together as a singing group. We learn dancing. We learn things like that. Next, they pick a number of students for the leadership team and the leadership team goes out for a week and they develop the outline for the play.

Like the program this young man described, we observed at several other HIV CBOs used “art therapy” to address emotional situations. These leisurely activities seemed to engage young people in meaningful ways, to avoid what key informants identified as “being seen as a number” or “just a person at-risk” for HIV.

Key informants believed the utility of “safe spaces” to derive not simply from their role in increasing HIV and STI testing rates, but also, crucially, because these spaces offered “important opportunities to socialize in a welcoming environment” and provided a platform to “talk about issues like racism and sexuality” and to “fight marginalization.” During interviews, service providers expressed a series of frustrations about current HIV funding strategies and priorities. Not only did they express concern about long-term decline in funding and staff for programs that supported safe spaces, but many felt that the preference among funders for specific kinds of programs, in particular individual level HIV testing programs, was misguided. For example, one physician explained, “a lot of service providers don’t like EBIs” (effective behavioral interventions) because they were “shortsighted” and “undervalued the staff’s work with outreach and community engagement, but that’s what’s funded.” Several CBO coordinators felt that programs that help clients find work and housing had more of an impact on their lives than testing, but that the emphasis on testing made these programs seem less important and less sustainable. Thus, without a commitment to policymaking and funding focused on capacity building to sustain safe spaces as integral to promoting HIV prevention, those working on the front-lines experience frustration and confusion about what they should prioritize with restricted funding.

Problems with funding restriction have led to program cuts that directly affect BMSM. One homeless 18-year-old gay man interviewed had been seriously affected when the drop-in center he attended had significantly reduced its opening hours. He felt that the administrators of the LGBT homeless drop-in center did not “know the common struggle of what the homeless youth does go through,” having been turned away with nowhere else to go.

For instance, department lock up and it might be a snowstorm outside. I'm like, "You're really gonna kick us all out? Like the kids who don't have places to go, and there's gonna be a snowstorm outside?" They told me that they don't have a lot of funding to pay staff for them to stay.

In participant observation at the drop-in center described above, one of the coordinators proudly showed that the institution was building a computer lab. When the lead ethnographer asked the coordinator how they could afford this but had less funding for staff-hours, he explained that this was a special project funded by a private company. Many of the youth at the drop-in center expressed confusion. They felt there was a contradiction that on the one hand the center had reduced its opening hours because of funding cuts, but on the other hand the center was expanding its computer lab. Overall then, the most salient barrier to maintaining safe spaces was restricted public funding for institutional infrastructure.

## Discussion

Safe spaces were viewed by both in-depth interviewees and key informants as essential to addressing social and economic issues that have been associated with HIV vulnerability, including institutional stigma [33], violence [34], lack of social support [35], housing instability [36], limited educational and limited employment opportunities [37]. Although several men attributed street violence to the lack of LGBT organizations in the outer boroughs of NYC compared to Manhattan, community stakeholders saw all of the boroughs as potentially dangerous, noting in particular concerns generated by recent attacks against feminine men and transgender women of color in New York City and throughout the United States [34]. Many BMSM preferred to discuss HIV as part of a larger set of social, economic and cultural issues, and some reacted negatively to public health campaigns that they perceived treated all BMSM as inherently at-risk because of their race and sexuality. These men found the emphasis on HIV among BMSM stigmatizing, and they resented how it obscured other struggles and dimensions of their lives. Our findings indicate that sustaining safe spaces, as a community-based approach, is one promising way to address the range of challenges that these BMSM felt were important in their lives. Through participant observation, we witnessed the ways in which brotherhoods and other men’s support groups enabled men to reflect on community perceptions of risk, and to think critically about how being perceived of as “at-risk” affects their engagement with prevention. Overall, these spaces offered a refuge from physical dangers and constituted enabling environments for confronting social and structural vulnerabilities.

In this way, safe spaces foster a mutually supportive environment, providing opportunity structures for health promotion [38–40]. The conceptual framework presented in Fig 2 summarizes different elements of safe spaces that respond to environmental vulnerabilities to HIV. The violence, discrimination, and social marginalization that men experienced were related to sexuality, feminine gender performance, and race. Most importantly, our study suggests that safe spaces are integral to the kinds of community-based approaches described by the National HIV/AIDS strategy. The literature on structural and environmental interventions provides insights into how changing people’s social context can be incorporated into interventions to promote healthy behaviors. In this paper, we emphasize the importance of environmental components to HIV interventions for mobilizing around a sense of community.

These findings are supported by recent meta-analyses and studies on HIV risk among BMSM [37,41], which have explicitly highlighted the need to address the structural and environmental drivers of HIV vulnerability. Thus, we argue that the kinds of community-engagement fostered by safe spaces ought to play a central part in efforts to address social marginalization as central to HIV prevention capacity. Despite their potential value as community-level resources for culturally-appropriate prevention programs, limited funding for CBOs and vertically-driven programmatic priorities threaten the sustainability of safe spaces at community-based organizations [42–45].

Community mobilization has been documented as critical to HIV prevention and treatment modalities in the history of the epidemic [32,46,47], and our study suggests that they continue to be essential to comprehensive prevention approaches. Taking structural and environmental challenges into account, some scholars have called for community engagement through “prevention literacy” programs, to address these structural and environmental conditions and to advance the acceptance of and adherence to new modalities of biomedical prevention [15,48]. Drawing on the legacy of treatment literacy campaigns that have educated populations throughout the world about HIV treatment, and have successfully fostered demand for treatment access among diverse populations, prevention literacy aspires to generate critical knowledge and also ownership of prevention modalities [15,21,48]. This kind of ownership has been most successful when generated within enabling environments that foster communication about broader social and structural conditions that challenge adherence to medication among particular populations [3,49]

In their study of community-based service providers working with BMSM, Saleh et al. (2011) emphasized the need to create “safe spaces” for BMSM [50]. Not only should “safe spaces” be included as part of interventions, but they are essential components of organizational capacity. But safe spaces have not yet been systematically incorporated into evidence-based interventions. Of the 33 effective behavioral HIV prevention interventions promoted by the CDC, MPowerment is the only intervention that explicitly incorporates safe spaces. Consistent with our findings, a recent evaluation of an MPowerment project in Detroit emphasized that the space was an essential aspect of the intervention [49], functioning both as a recruitment venue, and also as a space in which the men could pray, eat, hang out and find material and social support [49]. Other studies on the implementation of this EBI through the United States support the importance of safe spaces to program fidelity[29].

Our findings indicate that current funding structures are barriers to maintaining safe space. Thus this study suggests critical recommendations for HIV prevention interventions, especially for CBOs and those designing high impact HIV prevention programs. First, funding for institutional infrastructure for safe spaces should be separate from program-specific funding to improve the sustainability of an important aspect of community-based work. Both BMSM and community-stakeholders expressed that it was critical to have spaces that address violence, stigma, and socioeconomic vulnerabilities to improve HIV testing and engagement with care. Second, our study indicates that more emphasis on health promotion and safe environments for leisure in safe spaces would decrease the stigmatization of at-risk BMSM. Recognizing safe spaces in policy, such as the National Strategy, is critical for framing community-based approaches to HIV prevention among BMSM and other vulnerable groups (e.g., sex workers, injection drug users, women who suffer intimate partner violence).

## Supporting Information

S1 File Data Safe Spaces. (DOCX)
