# Supplementary material for: “You're Really Gonna Kick Us All Out?” Sustaining Safe Spaces for Community-Based HIV Prevention and Control among Black Men Who Have Sex with Men
Source: PLoS One. 2015 Oct 22;10(10):e0141326. doi: 10.1371/journal.pone.0141326 (PMC4619623; doi:10.1371/journal.pone.0141326)
Supplement: S1 File — (DOCX) [file pone.0141326.s001.docx]

| Age | Sex Identity. | Housing | Employm. | Ever Incarcer | No. Sex Partners  (6 mo.) | Narrative |
| --- | --- | --- | --- | --- | --- | --- |
| Private Insurance | | | | | | |
| 16 | Bisexual | Stable | None | No | 1 | “All the violent stuff that's going on nowadays is more stressful to me, like people crossing the street and beating up people for no reason because they gay or look gay. And that's been on the news quite often. I even told my boyfriend, because I know how feminine he walks and carry himself, "Be careful out there." And when I'm walking with him, I try to be careful too. I wouldn’t walk with him if he was in an area where it’s dangerous – called the projects.”  “In some parks, you have to time yourself to a certain degree where you wanna get out at a certain time because cops can't see you … And [men] take the risk of possibly being caught because they feel like they need to do whatever before they go wherever they travel, whether it’s home or to the club or from the club…. And sometimes that’s why it’s very important to kind of being able to assess the situation because… they will hurt you or attack you after.”  “So the police came up and they started just questioning what we were doing, why we were there. And of the people who were there, I felt singled out because I was visibly a person of color and the majority of them were not.”  “[The CBO] was a space where black men would meet and socialize with one another. We’d have these conversations, anything from politics or what’s happening specifically in the black gay or black homosexual community and just about being a bunch of brothers. And it age-varied. I think the oldest may have been 70, and the youngest may have been 18 or 19.”  “Having conversations with other people of color about their relationship to medicine usually is part of a larger conversation about racism and sexism in medicine and also in the legal system.”  “I’ve just gone to see if I have HIV or STDs. And I know it’s stigma with gay men, maybe even to this day that we worry because: If you're gay, you have AIDS. And if you don't have it, you will get it.” |
| 22 | Gay | Unstable | Temp | No | 1 |  |
| 22 | Gay | Stable | Fulltime | No | 2 |  |
| 31 | SGL/ Gay | Unstable | Fulltime | No | 40 |  |
| 32 | None | Stable | Fulltime | No | 5 |  |
| Medicaid | | | | | | |
| 17 | Gay | Unstable | Temp | Yes | 3 | “Negativity [that] comes with being homosexual from heterosexuals, from religion, from your grandma, from your aunt, from your uncle’s buddy, from your uncle – they could say everything about faggots and lesbians… It’s not supported in the black community; it’s definitely not supported through church.”  “My family believes that prayer heals everything. God will make a way. If you do the right thing and that wish or that prayer is to expect it, and this is what you feel you deserve. God heals— if you get sick, maybe you deserve this.”  “Was I went up there to the park, and I hooked up with this guy, and he kept saying let’s go over here. I kinda stopped ‘cause I saw somebody standing at the top of the stairs, and he was right behind me and pushed me. He said, son, where you going, and then all of these boys just came out of nowhere. And they was holding pipes and bats and all kinds of shit.”  “I was walkin’ home, and the cops they said I met somebody’s description, and it was like – I don’t know, in some way I feel like I made them come and, like, charge up against me because I – I was drunk. And when I get drunk, I’m very naïve. I say – Like, all right, he came up to me and I was like, “I don’t know what the fuck you’re talking about.”*…*And that’s when it went from there. I got maced. That was the worst feelin’ ever.”  “The cops stopped me and told me that I resemble a homicide suspect. And they searched me and then they asked me why I had so many singles in my pocket. And I told them that I was a waiter because I make tips. And he asked me what was I doing in this area and this and that. And I don't know. So that was another experience that I had… So my mother told me, ‘You should have asked them what was the homicide suspect? Was he black? So because you’re black you look like him?’”  “Well, because I got locked up my very first time going to Brooklyn. After that, I was like, "No. You will never catch me in Brooklyn."  I went to my first gay club. We were drinking. Someone got real crazy. Well, one of my friends that I was hanging out with got real crazy and stupid and decided to throw drinks across the room, thinking them shits could fly or something.”  “To get into Job Corps you have to be 16 to 25. Have to have no criminal record. You have to be willing to work. You can't be just coming out of jail and wanting to do Job Corps, no. Because it's a government based organization, governments don't help convicts right now.”  “It’s, again, socialization. It’s how you were taught about these things. You know what I mean? Minority and I know for the black community, the Caribbean community, Latinos, home remedies are a part of the culture, pretty much. You would hear growing up that pills are not good for you. They destroy your body, so on and so forth. So it would just be a part of you not to take medication.”  “But of course the doctors there were White… hey made me nervous a lot; I was always scared going there. I would always cry going there, because I was just young and nervous and didn’t actually know how to deal with the situation at the time. I was away from home and just a lot of different things; so yeah I was really nervous and it used to always be bad for me.”  “No, there was no primary care physician at all, you would just go to the emergency room.” |
| 17 | Gay | Homeless | None | No | 2 |  |
| 17 | Gay | Stable | None | No | 4 |  |
| 19 | Discreet Bisexual | Stable | Fulltime | No | 2 |  |
| 20 | Discreet Gay | Homeless | Temp | No | 1 |  |
| 21 | Bisexual | Stable | Temp | No | 2 |  |
| 21 | Gay | Stable | None | No | 1 |  |
| 24 | Gay | Stable | None | Yes | 3 |  |
| 24 | Gay | Unstable | Temp | Yes | 6 |  |
| 39 | Gay | Unstable | None | Yes | 3 |  |
| 45 | MSM | Stable | None | Yes | 5 |  |
| 46 | None/  Discreet | Stable | Fulltime | No | 3 |  |
| 41 | Gay | Stable | Fulltime | No | 10 |  |
| 48 | Straight | Stable | Temp | Yes | 5 |  |
| 45 | Straight | Unstable | None | Yes | 2 |  |
| 54 | Straight | Stable | None | Yes | 6 |  |
| 54 | Discreet Bisexual | Stable | Fulltime | Yes | 6 |  |
| Uninsured | | | | | | |
| 17 | Bisexual | Unstable | Temp | Yes | 20 | “It’s threatening to be with a feminine man… because you don't want people looking at you in a certain way. Stereotypes are for feminine men. They tend to be loud, obnoxious, ghetto, flamboyant, and just messy.”  Oh the Bronx is dangerous, Brooklyn is dangerous, oh, you know, gay bashing here and there all over the place, you know… Yeah, like I’ve heard more slurs; gay friends or associates of mine getting jumped or getting into fights, just all out brawls in Brooklyn.  “We all fight about not having equal rights because straight people are out here jumping and hate-criming us, but yet we’re in the clubs doing the same thing over petty stuff… And [the attacker was] masculine, you would never be able to tell he’s gay even though he has a boyfriend. So it was a bunch of grown gay men just attacking me.”  “I was like, "You know, I act pretty normal." I don't really call it normal, but that's the best word to use for it, like I'm not flamboyant, I'm not out in the open, I don't do anything to advertently put myself into danger because of my lifestyle, and stuff like that. I try to refrain from individuals that will put me into that arena….people who are just too loud…”  “Cops believe if you have condoms on you, you’re a sex worker. And we believe mostly in this community if you have condoms on you a lot, if you have condoms all over your house, that you’re a ho and that’s crazy we believe that in this community.”  “We do training, as in we do exercises, team building, skill building. We learn how to work together as a singing group. We learn dancing. We learn things like that. Next, they pick a number of students for the leadership team and the leadership team goes out for a week and they develop the outline for the play.”  “I'm like, "You're really gonna kick us all out? Like the kids who don't have places to go, and there's gonna be a snowstorm outside?" They told me that they don't have a lot of funding to pay staff for them to stay.”  “I was in this program. It's like a type of board where people sit down and they stand up for what they believe in. And there's two people that are representing it. They told us that they stop people. First, people for having condoms on them, which is crazy. I feel that why would you lock someone up for that unless you caught them doing it [sex work]? You know what I mean? Yeah, but they're not, so you just catch them with the condom. That's just really stupid. People need condoms.” |
| 18 | Gay | Homeless | None | No | 3 |  |
| 18 | Gay | Homeless | None | No | 3 |  |
| 21 | Gay | Homeless | None | No | 2 |  |
| 22 | Gay | Homeless | None | No | 3 |  |
| 26 | Gay | Stable | Temp | No | 30 |  |
| 27 | SGL | Unstable | None | No | 3 |  |
| 29 | Gay | Unstable | Fulltime | No | 7 |  |
| 47 | Discreet Gay | Unstable | Temp | No | 4 |  |
